# Prevalence of Metabolic Syndrome Associated with Body Burden Levels of Dioxin and Related Compounds among Japan’s General Population

**DOI:** 10.1289/ehp.0800012

**Published:** 2008-10-10

**Authors:** Hirokazu Uemura, Kokichi Arisawa, Mineyoshi Hiyoshi, Atsushi Kitayama, Hidenobu Takami, Fusakazu Sawachika, Satoru Dakeshita, Kentaro Nii, Hiroshi Satoh, Yoshio Sumiyoshi, Kenji Morinaga, Kazunori Kodama, Taka-ichiro Suzuki, Masaki Nagai, Tsuguyoshi Suzuki

**Affiliations:** 1 Department of Preventive Medicine, Institute of Health Biosciences, The University of Tokushima Graduate School, Tokushima, Japan;; 2 Study Group on the Accumulation of Dioxins in Humans, Tokyo, Japan

**Keywords:** cross-sectional study, metabolic syndrome, dioxins, PCBs, PCDDs, PCDFs, poly-chlorinated biphenyls, polychlorinated dibenzofurans, polychlorinated dibenzo-*p*-dioxins

## Abstract

**Background:**

Environmental exposure to some persistent organic pollutants has been reported to be associated with a metabolic syndrome in the U.S. population.

**Objectives:**

We evaluated the associations of body burden levels of dioxins and related compounds with the prevalence of metabolic syndrome among the general population in Japan.

**Methods:**

We conducted a cross-sectional study with 1,374 participants not occupationally exposed to these pollutants, living throughout Japan during 2002–2006. In fasting blood samples, we measured biochemical factors and determined lipid-adjusted concentrations of 10 polychlorinated dibenzo-*p*-dioxins (PCDDs), 7 polychlorinated dibenzofurans (PCDFs), and 12 dioxin-like poly-chlorinated biphenyls (DL-PCBs) all of which have toxic equivalency factors. We also performed a questionnaire survey.

**Results:**

The toxic equivalents (TEQs) of PCDDs, PCDFs, and DL-PCBs and total TEQs had significant adjusted associations with metabolic syndrome, whether or not we excluded diabetic subjects. By analyzing each component of metabolic syndrome separately, the DL-PCB TEQs and total TEQs were associated with all components, and the odds ratios (ORs) in the highest quartile of DL-PCB TEQs in four of the five components were higher than those for PCDDs or PCDFs. We also found congener-specific associations with metabolic syndrome; in particular, the highest quartiles of PCB-126 and PCB-105 had adjusted ORs of 9.1 and 7.3, respectively.

**Conclusions:**

These results suggest that body burden levels of dioxins and related compounds, particularly those of DL-PCBs, are associated with metabolic syndrome. Of the components, high blood pressure, elevated triglycerides, and glucose intolerance were most closely associated with these pollutants.

Polychlorinated dibenzo-*p*-dioxins (PCDDs) and polychlorinated dibenzofurans (PCDFs), collectively called dioxins, are produced through the burning of garbage and some types of chemical manufacturing processes. Legal regulations of dioxins in the 1990s in Japan have achieved a 95% reduction in their emissions from incinerators (Ministry of the Environment, Government of Japan 2004). Some forms of polychlorinated biphenyls (PCBs), which are persistent organic pollutants (POPs), exhibit toxic actions similar to 2,3,7,8-tetra chlorodibenzo-*p*-dioxin (TCDD) and are called dioxin-like PCBs (DL-PCBs). PCDDs/PCDFs and DL-PCBs are widely persistent in the environment because they are lipophilic and resistant to biological and chemical degradation. Several congeners of these pollutants exhibit various biological and toxic actions, such as dermal toxicity, immunotoxicity, carcinogenicity, and adverse effects on reproductive, neuro behavioral, and endocrine functions (Lindström et al. 1995; Weisglas-Kuperus 1998). Johnson et al. (2001) and Lee et al. (2006) have suggested that these actions could be induced by low-level exposure to these pollutants.

Metabolic syndrome, characterized by a cluster of metabolic disorders including central obesity, glucose intolerance, dyslipidemia, and hypertension, has been increasing in developed countries (Flegal et al. 2002; Ford 2005). The health impact of each component of the metabolic syndrome may be small, but the co-occurrence of several components may have considerably greater impact than any one alone (Nakanishi et al. 2003). Subjects suffering from metabolic syndrome may be at increased risk of type 2 diabetes (Lorenzo et al. 2003; Sattar et al. 2003) and cardiovascular diseases (Lakka et al. 2002; Malik et al. 2004; McNeill et al. 2005). Recently, background environmental exposure to some POPs has been reported to be associated with increased risk of diabetes (Lee et al. 2006) and with increased risk of metabolic syndrome (Lee et al. 2007) in the U.S. population in the 1999–2002 National Health and Nutrition Examination Survey (NHANES). Metabolic syndrome has become widely preva lent in the populations in Asian countries, including Japan, but whether similar associations exist among Asian populations has not been evaluated. A survey on the accumulation of dioxins and related compounds in humans was carried out beginning in 2002 under the supervision of the Ministry of the Environment, Government of Japan. In this article, we report the recent 5-year results of a cross-sectional study to evaluate the associations of body burden levels of PCDDs, PCDFs, and DL-PCBs with the prevalence of metabolic syndrome, and with the prevalence of its components, among general inhabitants in Japan.

## Materials and Methods

### Survey areas and participants

We conducted this survey from 2002 to 2006 in Japan on 1,374 participants (627 male and 747 female) 15–73 years of age. The selection of survey areas and the recruitment of the participants have been reported previously (Uemura et al. 2008). Briefly, we divided the whole of Japan into five regional blocks (Table 1) and selected a single prefecture from each regional block every survey year. We recruited approximately 50 individuals in each prefecture; approximately 20 were from urban areas, 15 from farming village areas, and 15 from fishing village areas, with almost equal distribution of age and sex among the three areas. As a whole, the subjects participated from 75 different residential areas of 25 prefectures through 5 survey years and were evenly distributed in age and sex among the urban, farming, and fishing village areas in each survey year. Participants were required to have been living in the same area for at least 10 years, to have had no known occupational exposure to PCDDs/PCDFs and DL-PCBs, and to have no known severe anemia or other health problems that interfere with blood sampling. Participation in this study was essentially voluntary, and after we explained the details of this survey, we obtained written informed consent from each participant. We designed the study to recruit at least 250 participants every survey year; few subjects were excluded because of known severe anemia or other health problems during the survey years. The study protocol was reviewed and approved by the Ethical Committee of the Ministry of the Environment, Government of Japan.

### Questionnaire

We asked participants to complete a questionnaire to obtain data on individual characteristics, including body height and weight, residential and occupational histories, smoking and drinking habits, and past history of diseases and treatments. Regarding drinking habits, subjects were asked how often they had consumed beer, sake, shochu (rough distilled spirits), and whisky over the previous month. The frequency of each item in the questionnaire was classified into five categories: almost everyday (6 times/week), 3–4 times/week (3.5/week), 1–2 times/week (1.5/week), 1–2 times/month (0.35/week), and almost never (0.1/week). We did not specify the unit or portion of each item. The frequency of alcohol consumption was summarized as the sum of the frequency of each item and then divided into three categories: regular, ≥ 6 times/week; often, 1.5 to < 6 times/week; rarely or never, < 1.5 times/week.

### Measurements

We obtained approximately 25 mL fasting venous blood from each participant, with 20 mL collected into Vacutainer tubes containing sodium heparin (VT-100H, Terumo, Tokyo, Japan; or 367677, Becton Dickinson and Company, Tokyo, Japan). PCDDs, PCDFs, and DL-PCBs were analyzed in whole blood at the Institute of General Science for the Environment, METOCEAN Environment (currently IDEA Consultants, Inc., Shizuoka, Japan) by isotope dilution high-resolution gas chromatography/mass spectrometry, after liquid/liquid extraction and gel cleanup. The detailed analytical procedure of these chemicals has been previously reported (Nakamura et al. 2008). Percent blood lipid was measured gravimetrically using a sulfuric ammonium-ethanol/hexane technique. Concentrations of 7 PCDD congeners, 10 PCDF congeners, and 12 DL-PCB congeners that have toxic equivalency factors (TEFs). The limit of detection (LOD) was 1 pg/g lipid for PCDDs/PCDFs with four or five chlorine atoms, 2 pg/g lipid for PCDDs/PCDFs with six or seven chlorine atoms, 4 pg/g lipid for PCDDs/PCDFs with eight chlorine atoms, and 10 pg/g lipid for DL-PCBs.

We calculated toxic equivalents (TEQs) using the 1998 World Health Organization TEF (Van den Berg et al. 1998). We assigned zero concentrations to pollutant levels < LOD.

We determined high-density lipoprotein (HDL) cholesterol, triglycerides, and hemoglobin A1c (HbA1c) in the blood with an automatic biochemical analyzer (model 7450; Hitachi, Tokyo, Japan).

Blood pressure was measured in each subject sitting at rest. If a measurement was extremely high or low or far from everyday values, we tried again after a short rest and used the second measurement as the final measurement.

### Assessment of metabolic syndrome

We assessed the prevalence of metabolic syndrome using a modification of the National Cholesterol Education Program (NCEP) Adult Treatment Panel III (ATP III) definition (NCEP 2002). Some studies in Asia have suggested that the criteria of central obesity proposed by the NCEP, which is based on the data from Caucasians, is inappropriate for Asian populations, whose builds are generally smaller than those of Caucasians (Ko et al. 2005; Tan et al. 2004). Therefore, we diagnosed subjects with metabolic syndrome when they satisfied three or more of the following five criteria: *a*) body mass index (BMI) ≥ 25 kg/m^2^ (rather than by abdominal waist circumference); *b*) serum triglycerides ≥ 150 mg/dL; *c*) serum HDL < 40 mg/dL in men or < 50 mg/dL in women; *d*) systolic blood pressure ≥ 130 mmHg and/or diastolic blood pressure ≥ 85 mmHg, or self-reported history of physician-diagnosed hypertension; *e* ) HbA1c ≥ 5.6% (rather than fasting serum glucose), or self-reported history of physician-diagnosed diabetes.

The association of diabetes mellitus with PCBs and other POPs is well established, and diabetes mellitus is a partial component of metabolic syndrome. Therefore, we further investigated the associations of these pollutants with metabolic syndrome in the absence of diabetes mellitus by excluding subjects who either self-reported a history of physician-diagnosed diabetes or had plasma HbA1c > 6.1%. This HbA1c level predicts fasting plasma glucose ≥ 126 mg/dL, the standard for determining diabetes, with a sensitivity of 63.2% and a specificity of 97.4% (Rohlfing et al. 2000).

### Statistical analysis

We evaluated the sex difference of BMIs by the Wilcoxon rank-sum test. We analyzed the associations of the TEQs of PCDDs, PCDFs, DL-PCBs, and total TEQs with the prevalence of metabolic syndrome by excluding or including prevalent diabetes in logistic regressions, nonadjusted and adjusted for age, sex, smoking and drinking habits, regional block, residential area, and survey year. We tested the adjusted associations of the TEQs with the five components of metabolic syndrome in logistic regressions. We also evaluated separately the adjusted associations of the concentrations of the 16 selected congeners for which > 75% of the subjects had concentrations > LOD with the prevalence of metabolic syndrome in logistic regressions: 1,2,3,7,8-penta (Pe) CDD, 1,2,3,6,7,8-hexa (Hx) CDD, 1,2,3,4,6,7,8-hepta (Hp) CDD, octaCDD (OCDD), 2,3,4,7,8-PeCDF, 1,2,3,6,7,8-HxCDF, PCB-126, PCB-169, PCB-105, PCB-114, PCB-118, PCB-123, PCB-156, PCB-157, PCB-167, and PCB-189. In these analyses, we defined the first quartile (< 25th percentile) as the reference; created dummy variables for sex, regional block, residential area, survey year, smoking habit, and drinking habit; and included all except reference categories in the model. We conducted tests of trend using the median value for each quartile of the TEQs or the concentrations of the congeners in logistic models. All statistical analyses were performed using SAS software (version 8.2; SAS Institute Inc., Cary, NC, USA). All *p*-values are two-tailed, and we considered those < 0.05 statistically significant.

## Results

The baseline characteristics of the participants are shown in Table 1. Of the 1,374 subjects, 627 (45.6%) were male and 747 (54.4%) were female. The medians (25th–75th percentiles) of BMI were 23.4 (21.6–25.7) kg/m^2^ and 21.6 (20.0–23.9) kg/m^2^ in male and female subjects, respectively, with a significant difference (*p* < 0.001). Table 2 shows the prevalence of metabolic syndrome and its five components in the subjects. Of the 1,374 subjects, we defined 160 (11.6%) subjects as having metabolic syndrome (105 men, 55 women). High blood pressure had a higher prevalence than the other components.

Table 3 lists the concentrations and TEQs for each PCDD, PCDF, and DL-PCB congener in the blood of the subjects. The medians of the TEQs of PCDDs, PCDFs, DL-PCBs, and total TEQs were 7.4, 4.5, 7.6, and 20 pg TEQ/g lipid, respectively.

Table 4 lists the nonadjusted and adjusted associations of the TEQs with the prevalence of metabolic syndrome. All of the TEQs for PCDDs, PCDFs, and DL-PCBs and total TEQs had significant nonadjusted and adjusted associations with the prevalence of metabolic syndrome (tests of trend were all significant). The highest quartile of total TEQs had a high adjusted odds ratio (OR) and 95% confidence interval (CI) of 5.3 (2.3–13) compared with the referent. Table 5 lists the nonadjusted and adjusted associations of the TEQs with the prevalence of metabolic syndrome, excluding the subjects with prevalent diabetes. Among the 65 diabetic subjects, we diagnosed 38 subjects with metabolic syndrome. When we excluded prevalent diabetes from the analyses, we defined 122 subjects as having metabolic syndrome. The adjusted associations of the TEQs of DL-PCBs with metabolic syndrome were strengthened, whereas those of PCDFs were slightly attenuated (*p* for trend = 0.07).

The adjusted associations of the TEQs with the prevalence of each component of metabolic syndrome are shown in Table 6. All of the TEQs of PCDDs, PCDFs, and DL-PCBs and total TEQs were associated with high blood pressure and elevated triglycerides (tests of trend were all significant). The TEQs of PCDDs and DL-PCBs and total TEQs showed trends for the associations with high HbA1c (*p* for trends all < 0.01). In particular, the highest quartiles of DL-PCBs and total TEQs had adjusted ORs of 8.0 and 8.6, respectively, in the prevalence of high HbA1c. Of note, the TEQ of DL-PCBs as well as total TEQs showed trends for the associations with all five components of metabolic syndrome, and the ORs in the highest quartile of the TEQ of DL-PCBs in four of the five components were higher than those for PCDDs or PCDFs. In contrast, the TEQs of PCDFs were not associated with high BMI (*p* for trend = 0.56) or low HDL (*p* for trend = 0.12).

Table 7 lists the adjusted associations of concentrations of selected congeners with the prevalence of metabolic syndrome. We found significant trends for associations with metabolic syndrome for 1,2,3,7,8-PeCDD, 1,2,3,6,7,8-HxCDD, 1,2,3,4,6,7,8-Hp-CDD, OCDD, 2,3,4,7,8-PeCDF, PCB-126, PCB-105, PCB-114, PCB-118, PCB-123, and PCB-167. The highest quartiles of PCB-126 and PCB-105 had adjusted ORs (95%-CI) of 9.1-(4.1–21) and 7.3-(3.4–17), respectively, compared with the respective referents.

## Discussion

Several studies of veterans exposed to high levels of dioxins have suggested that one of the plausible diseases associated with dioxins is type 2 diabetes (Henriksen et al. 1997; Michalek et al. 1999). This association is supported by similar epidemiologic studies in subjects exposed to high levels of dioxins in Seveso, Italy (Pesatori et al. 2003), and Korean Vietnam veterans exposed to Agent Orange (Kim et al. 2003). However, a causal association of dioxins or other POPs with diabetes is far from established. Recently, low levels of exposure to dioxins were reported to be associated with increased risk of diabetes (Lee et al. 2006), and additionally with increased risk of metabolic syndrome (Lee et al. 2007), in the U.S. population in the 1999–2002 NHANES. Metabolic syndrome has been increasing in Asian countries, including Japan, with the Westernization of lifestyle. We studied whether associations similar to those found in the U.S. population exist among the Japanese population. Lee et al. (2007) investigated the associations between some POPs and metabolic syndrome among nondiabetic subjects. However, excluding diabetic subjects could result in a sample less representative of general inhabitants. Therefore, in the present study we examined the associations both including and excluding the diabetic subjects.

We found that all of the TEQs of PCDDs, PCDFs, and DL-PCBs and total TEQs had significant nonadjusted and adjusted associations with the prevalence of metabolic syndrome. The highest quartile of total TEQs had a high adjusted OR of 5.3. Analyses of each component of metabolic syndrome indicated that all of the TEQs of PCDDs, PCDFs, and DL-PCBs and total TEQs were associated with high blood pressure and elevated triglycerides. The TEQs of PCDDs and DL-PCBs and total TEQs showed significant trends for associations with high HbA1c. In particular, the highest quartiles of DL-PCB TEQs and total TEQs had high adjusted ORs of 8.0 and 8.6, respectively, in the prevalence of high HbA1c. Also, the DL-PCB TEQs and total TEQs were associated with all five components of metabolic syndrome, and the ORs in the highest quartile of the TEQ of DL-PCBs in four of the five components were higher than those for PCDDs or PCDFs. From these findings, of the five components of metabolic syndrome, high blood pressure, elevated triglycerides, and glucose intolerance may be most closely associated with these pollutants, particularly with DL-PCBs. In contrast, high BMI was not associated with TEQs except for DL-PCBs. Of the five components, high BMI might be the least associated with exposure to these pollutants.

TEQ-based analyses can be justified if the aryl hydrocarbon (Ah) receptor is the mechanism involved. PCDDs/PCDFs and DL-PCBs that have been assigned a TEF are known to exhibit various biological and toxic actions through binding with Ah receptors, so we conducted the TEQ-based analyses. However, Lee et al. (2007) recently reported that the background exposure to some POPs may be closely related to metabolic syndrome, with different POPs related to different metabolic syndrome traits. To examine whether Ah-receptor–mediated mechanisms of the examined pollutants are critical in their link to metabolic syndrome, we further analyzed the associations of the concentration of each congener separately with the prevalence of metabolic syndrome. We found that 12 of the selected 16 congeners, widely distributed among PCDDs, PCDFs, and DL-PCBs, had trends for association with metabolic syndrome. In particular, the highest quartiles of PCB-126 and PCB-105 had adjusted ORs of 9.1 and 7.3, respectively, a finding consistent with that of the TEQ-based analyses.

We found associations of PCDD and PCDF TEQs with the prevalence of metabolic syndrome; in contrast, Lee et al. (2007) found no such associations. These associations in our TEQ-based analyses agreed with the results of our congener-specific analyses. Lee et al. (2007) also reported that the risk of metabolic syndrome was highest in the third quartile and then plateaued in the case of DL-PCBs, whereas we found a significant trend for the association between the TEQ of DL-PCBs and metabolic syndrome. This association in our study persisted or strengthened when diabetic subjects were excluded from the analyses. Although we could not clearly explain this discrepancy between the findings of Lee et al. (2007) and ours, the difference in prevalence of metabolic syndrome (24.3% and 9.3%, respectively, when excluding diabetic subjects) might influence this discrepancy.

The associations observed in this cross-sectional study should be carefully interpreted regarding cause–effect relations. For diabetic subjects, slower elimination of dioxins was not supported in a study on Vietnam veterans (Michalek et al. 2003), in which no difference in TCDD half-life was reported between diabetic and nondiabetic subjects. However, for subjects with metabolic syndrome, elimination half-life of dioxins has not been investigated. Some previous studies have shown that individuals with higher BMI, which was one component of metabolic syndrome in our study, have reduced dioxin clearance (Blanck et al. 2000; Flesch-Janys et al. 1996). Therefore, suffering from metabolic syndrome might impair the metabolism or the excretion of these pollutants and might lead to a greater accumulation in the body. Further prospective studies will be needed to clarify this causality.

Metabolic syndrome has recently become prevalent, whereas the body burden levels of PCDDs/PCDFs and DL-PCBs have a decreasing trend (Konishi et al. 2001; Porta 2006; Wittsiepe et al. 2000). Therefore, the dose–response relations of the TEQs or the concentrations of these pollutants in the blood with metabolic syndrome observed in this study or another (Lee et al. 2007) seem to conflict. One possible explanation is that toxic actions of these pollutants may be strengthened in obesity. This explanation is supported by a recent molecular epidemiologic study of diabetogenic effects of dioxin exposure, which showed an inverse correlation between the ratio of GLUT4 to nuclear factor kappa B and serum dioxin concentrations in obesity and family history of diabetes (Fujiyoshi et al. 2006). To clarify this hypothesis in our data, we further analyzed the associations between the concentrations of these pollutants and the prevalence of each component of metabolic syndrome except BMI after stratifying by BMI (≥ 25 kg/m^2^ and < 25 kg/m^2^). However, the associations did not show expected tendencies (data not shown), so the reliability of this hypothesis should be further verified. Another explanation is that PCDDs/PCDFs or DL-PCBs contribute to the occurrence of metabolic syndrome, but other factors including the Westernization of lifestyle also affect the occurrence of metabolic syndrome.

The strength of the present study is that our subjects represent the general population of Japan. This study has several limitations. First, as stated above, the findings in this study should be interpreted with caution regarding cause–effect relationships because of the cross-sectional design. Second, we diagnosed metabolic syndrome using a criterion of BMI instead of waist circumference, but BMI may not rigorously reflect central obesity. Similarly, we used HbA1c as a criterion instead of fasting serum glucose because we did not have data on fasting serum glucose.

In conclusion, this representative data among the general population of Japan showed significant associations of body burden levels of dioxins and related compounds with the prevalence of metabolic syndrome. Of the five components of metabolic syndrome, high blood pressure, elevated triglycerides, and glucose intolerance may be closely associated with the blood levels of these pollutants, especially with DL-PCBs. Basic studies in animals and further epidemiologic studies using longitudinal designs should be further conducted to define the mechanism and the causality between exposure to these pollutants and metabolic syndrome.

## Figures and Tables

**Table 1:** Baseline characteristics (%) of the participants.

	Both sexes (*n* = 1,374)	Male (*n* = 627)	Female (*n* = 747)
Age (years)			
15–29	20.7	22.7	19.0
30–39	18.7	20.6	17.1
40–49	21.9	20.3	23.3
50–59	24.0	23.3	24.6
60–73	14.7	13.2	15.9
Regional block			
Hokkaido/Tohoku	19.9	19.1	20.5
Kanto/Koshin’etsu	20.0	17.2	22.4
Tokai/Hokuriku/Kinki	20.5	24.4	17.3
Chugoku/Shikoku	19.3	21.2	17.7
Kyushu/Okinawa	20.3	18.0	22.2
Residential area			
Urban	40.2	37.6	42.3
Farming village	32.0	33.0	31.2
Fishing village	27.8	29.4	26.5
Survey year			
2002	18.9	18.7	19.0
2003	19.8	19.6	20.0
2004	19.2	17.9	20.4
2005	21.0	21.5	20.5
2006	21.2	22.3	20.2
Smoking habit			
Current	21.8	40.2	6.3
Past	13.1	22.0	5.6
Never	64.6	37.5	87.4
Unknown	0.5	0.3	0.7
Drinking habit			
Regular	20.7	36.8	7.1
Often	22.3	27.3	18.2
Rarely or never	53.7	32.7	71.4
Unknown	3.3	3.2	3.3

**Table 2:** Prevalence of metabolic syndrome and its five components in the participants (%).

	Both sexes (*n* = 1,374)	Male (*n* = 627)	Female (*n* = 747)
Metabolic syndrome			
Yes	11.6	16.7	7.4
No	87.0	81.5	91.6
Unknown	1.4	1.8	1.1
Components^a^			
BMI ≥ 25 kg/m ^2^			
Yes	23.7	32.4	16.5
No	75.3	66.2	82.9
Unknown	1.0	1.4	0.7
Blood pressure ≥ 130/85 mmHg or a history of physician-diagnosed hypertension			
Yes	41.3	50.4	33.7
No	58.6	49.6	66.1
Unknown	0.1	0.0	0.1
Triglycerides ≥ 150 mg/dL			
Yes	16.2	26.3	7.8
No	83.5	73.4	92.0
Unknown	0.3	0.3	0.3
HDL cholesterol < 40 mg/dL in men or 50 mg/dL in women			
Yes	10.3	10.4	10.3
No	89.4	89.3	89.4
Unknown	0.3	0.3	0.3
HbA1c ≥ 5.6% or a history of physician-diagnosed diabetes			
Yes	11.2	13.4	9.4
No	88.8	86.6	90.6
Unknown	0.0	0.0	0.0

a We diagnosed metabolic syndrome as the presence of three or more of these five components.

**Table 3:** Concentrations and TEQs for PCDD, PCDF, and DL-PCB congener in the blood of the subjects.

Congener	Concentration [pg/g lipid; median (25%–75%)]	TEF^a^	TEQ [pg TEQ/g lipid; median (25%–75%)]
PCDDs			
2,3,7,8-TCDD	1.0 (0.0–2.0)	1	1.0 (0.0–2.0)
1,2,3,7,8-PeCDD	5.0 (3.0–7.0)	1	5.0 (3.0–7.0)
1,2,3,4,7,8-HxCDD	0.0 (0.0–3.0)	0.1	0.0 (0.0–0.30)
1,2,3,6,7,8-HxCDD	15 (9.0–21)	0.1	1.5 (0.90–2.1)
1,2,3,7,8,9-HxCDD	3.0 (0.0–4.0)	0.1	0.30 (0.0–0.40)
1,2,3,4,6,7,8-HpCDD	12 (9.0–18)	0.01	0.12 (0.09–0.18)
OCDD	140 (89–230)	0.0001	0.01 (0.01–0.02)
Total PCDDs			7.4 (4.6–11.2)

PCDFs			
2,3,7,8-TCDF	1.0 (0.0–2.0)	0.1	0.10 (0.0–0.20)
1,2,3,7,8-PeCDF	0.0 (0.0–0.0)	0.05	0.0 (0.0–0.0)
2,3,4,7,8-PeCDF	7.0 (5.0–11.0)	0.5	3.5 (2.5–5.5)
1,2,3,4,7,8-HxCDF	3.0 (0.0–4.0)	0.1	0.30 (0.0–0.40)
1,2,3,6,7,8-HxCDF	3.0 (2.0–5.0)	0.1	0.30 (0.20–0.50)
1,2,3,7,8,9-HxCDF	0.0 (0.0–0.0)	0.1	0.0 (0.0–0.0)
2,3,4,6,7,8-HxCDF	0.0 (0.0–2.0)	0.1	0.0 (0.0–0.20)
1,2,3,4,6,7,8-HpCDF	2.0 (0.0–3.0)	0.01	0.02 (0.0–0.03)
1,2,3,4,7,8,9-HpCDF	0.0 (0.0–0.0)	0.01	0.0 (0.0–0.0)
OCDF	0.0 (0.0–0.0)	0.0001	0.0 (0.0–0.0)
Total PCDFs			4.5 (2.9–6.8)
Total PCDDs/PCDFs			12 (7.7–18)

PCBs			
PCB-77	0.0 (0.0–0.0)	0.0001	0.0 (0.0–0.0)
PCB-81	0.0 (0.0–0.0)	0.0001	0.0 (0.0–0.0)
PCB-126	40 (22–78)	0.1	4.0 (2.2–7.8)
PCB-169	30 (20–50)	0.01	0.30 (0.20–0.50)
Non-*ortho* PCBs			4.4 (2.4–8.3)
PCB-105	1,300 (750–2,400)	0.0001	0.13 (0.08–0.24)
PCB-114	460 (250–830)	0.0005	0.23 (0.13–0.42)
PCB-118	7,500 (4,200–13,000)	0.0001	0.75 (0.42–1.3)
PCB-123	100 (60–200)	0.0001	0.01 (0.01–0.02)
PCB-156	2,800 (1,500–4,800)	0.0005	1.4 (0.75–2.4)
PCB-157	770 (420–1,300)	0.0005	0.39 (0.21–0.65)
PCB-167	1,300 (730–2,300)	0.00001	0.01 (0.01–0.02)
PCB-189	320 (180–580)	0.0001	0.03 (0.02–0.06)
Mono-*ortho* PCBs			3.0 (1.6–5.1)
Total DL-PCBs			7.6 (4.4–13)
Total TEQs			20 (12–31)

TCDF, tetrachlorodibenzofuran.

a Data from Van den Berg et al. (1998).

**Table 4:** Nonadjusted and adjusted associations of the TEQs of PCDDs, PCDFs, DL-PCBs and total TEQs with the prevalence of metabolic syndrome.

			OR (95% CI)	
TEQs	No. of subjects	No. of cases	Nonadjusted	Adjusted^a^
PCDDs				
< 4.60	343	15	Referent	Referent
≥ 4.60 to < 7.39	344	39	2.8 (1.5–5.4)	2.2 (1.2–4.4)
≥ 7.39 to < 11.20	339	38	2.7 (1.5–5.2)	2.1 (1.1–4.3)
≥ 11.20	348	68	5.4 (3.1–10)	3.2 (1.6–6.7)
*p* for trend			< 0.01	< 0.01

PCDFs				
< 2.90	330	9	Referent	Referent
≥ 2.90 to < 4.50	347	40	4.6 (2.3–10)	4.0 (1.9–9.3)
≥ 4.50 to < 6.80	352	48	5.6 (2.9–12)	4.1 (1.9–9.7)
≥ 6.80	345	63	8.0 (4.1–17)	4.4 (2.0–11)
*p* for trend			< 0.01	0.04

DL-PCBs				
< 4.40	339	14	Referent	Referent
≥ 4.40 to < 7.60	339	27	2.0 (1.1–4.1)	1.9 (0.95–4.0)
≥ 7.60 to < 13.00	325	39	3.2 (1.7–6.2)	2.8 (1.3–6.2)
≥ 13.00	371	80	6.4 (3.6–12)	4.8 (2.2–11)
*p* for trend			< 0.01	< 0.01

Total TEQs				
< 12.00	303	10	Referent	Referent
≥ 12.00 to < 20.00	363	29	2.6 (1.3–5.6)	2.3 (1.1–5.3)
≥ 20.00 to < 31.00	353	47	4.5 (2.3–9.7)	3.7 (1.7–8.7)
≥ 31.00	355	74	7.7 (4.1–16)	5.3 (2.3–13)
*p* for trend			< 0.01	< 0.01

a Adjusted for age, sex, smoking habit, drinking habit, regional block, residential area, and survey year (model df = 19).

**Table 5:** Nonadjusted and adjusted associations of the TEQs of PCDDs, PCDFs, DL-PCBs and total TEQs with the prevalence of metabolic syndrome, excluding the subjects with prevalent diabetes.

			OR (95% CI)	
TEQs	No. of subjects	No. of cases	Nonadjusted	Adjusted^a^
PCDDs				
< 4.49	325	12	Referent	Referent
≥ 4.49 to < 7.27	329	31	2.7 (1.4–5.6)	2.2 (1.1–4.8)
≥ 7.27 to < 11.00	311	28	2.6 (1.3–5.3)	2.1 (0.99–4.7)
≥ 11.00	344	51	4.6 (2.5–9.2)	3.4 (1.6–7.6)
*p* for trend			< 0.01	< 0.01

PCDFs				
< 2.83	326	9	Referent	Referent
≥ 2.83 to < 4.40	326	31	3.7 (1.8–8.3)	3.5 (1.6–8.2)
≥ 4.40 to < 6.60	323	37	4.5 (2.2–10)	3.8 (1.7–9.2)
≥ 6.60	334	45	5.5 (2.8–12)	3.8 (1.6–9.7)
*p* for trend			< 0.01	0.07

DL-PCBs				
< 4.28	327	9	Referent	Referent
≥ 4.28 to < 7.40	320	24	2.9 (1.4–6.7)	3.1 (1.4–7.4)
≥ 7.40 to < 12.87	334	35	4.2 (2.1–9.4)	5.0 (2.1–13)
≥ 12.87	328	54	6.9 (3.5–15)	7.3 (2.9–20)
*p* for trend			< 0.01	< 0.01

Total TEQs				
< 12.00	303	10	Referent	Referent
≥ 12.00 to < 19.00	318	22	2.2 (1.0–4.9)	2.2 (0.98–5.0)
≥ 19.00 to < 30.00	345	35	3.3 (1.7–7.2)	3.2 (1.4–7.6)
≥ 30.00	343	55	5.6 (2.9–12)	5.1 (2.1–13)
*p* for trend			< 0.01	< 0.01

a Adjusted for age, sex, smoking habit, drinking habit, regional block, residential area, and survey year (model df = 19).

**Table 6:** Adjusted ORs (95% CIs) of the prevalence of each component of metabolic syndrome by quartiles of the TEQs of PCDDs, PCDFs, and DL-PCBs and total TEQs (quartile 1 is the referent).

Component	Quartile 2	Quartile 3	Quartile 4	*p* for trend
BMI ≥ 25 kg/m^2^				
PCDDs	1.5 (0.97–2.2)	1.5 (0.93–2.3)	1.5 (0.91–2.4)	0.30
PCDFs	1.3 (0.85–2.0)	1.5 (0.97–2.4)	1.3 (0.79–2.2)	0.56
DL-PCBs	1.7 (1.1–2.6)	1.8 (1.1–3.0)	2.6 (1.5–4.7)	< 0.01
Total TEQs	1.3 (0.86–2.1)	1.9 (1.2–3.1)	1.9 (1.1–3.3)	0.07

Blood pressure ≥ 130/85 mmHg or a history of physician-diagnosed hypertension				
PCDDs	0.99 (0.68–1.4)	1.0 (0.70–1.6)	1.6 (1.0–2.5)	0.01
PCDFs	1.3 (0.92–2.0)	1.6 (1.1–2.4)	1.9 (1.2–3.0)	< 0.01
DL-PCBs	1.0 (0.71–1.6)	1.1 (0.69–1.7)	1.9 (1.1–3.1)	< 0.01
Total TEQs	1.3 (0.86–1.9)	1.2 (0.81–1.9)	1.9 (1.1–3.1)	< 0.01

Triglycerides ≥ 150 mg/dL				
PCDDs	2.1 (1.3–3.5)	2.1 (1.2–3.7)	2.7 (1.5–4.8)	< 0.01
PCDFs	1.5 (0.87–2.5)	2.1 (1.2–3.6)	2.2 (1.2–4.1)	0.02
DL-PCBs	2.4 (1.4–4.3)	3.4 (1.8–6.6)	5.2 (2.6–11)	< 0.01
Total TEQs	2.0 (1.1–3.5)	3.0 (1.6–5.6)	3.8 (1.9–7.5)	< 0.01

HDL cholesterol < 40 mg/dL in males or 50 mg/dL in females				
PCDDs	1.5 (0.83–2.8)	1.8 (0.93–3.4)	3.2 (1.7–6.4)	< 0.01
PCDFs	1.5 (0.85–2.8)	2.0 (1.0–3.7)	1.9 (0.98–4.0)	0.12
DL-PCBs	1.1 (0.58–1.9)	1.9 (0.98–3.8)	2.1 (0.98–4.5)	0.06
Total TEQs	1.3 (0.72–2.5)	1.9 (0.97–3.8)	2.7 (1.3–5.9)	< 0.01

HbA1c ≥ 5.6% or a history of physician-diagnosed diabetes				
PCDDs	2.7 (1.2–6.6)	3.5 (1.6–8.7)	4.6 (2.0–12)	< 0.01
PCDFs	2.1 (0.93–5.2)	3.9 (1.8–9.6)	3.2 (1.4–8.3)	0.06
DL-PCBs	2.1 (0.90–5.3)	3.1 (1.3–8.0)	8.0 (3.2–22)	< 0.01
Total TEQs	3.3 (1.3–10)	4.5 (1.7–14)	8.6 (3.1–28)	< 0.01

Adjusted for age, sex, smoking habit, drinking habit, regional block, residential area, and survey year (model df = 19).

**Table 7:** Adjusted ORs (95% CIs) of the prevalence of metabolic syndrome by quartiles of the concentrations of the selected congeners (Q1 = referent).

Congener	Quartile 2	Quartile 3	Quartile 4	*p* for trend
PCDDs				
1,2,3,7,8-PeCDD	2.8 (1.2–8.4)	2.0 (0.77–6.4)	3.7 (1.4–12)	0.04
1,2,3,6,7,8-HxCDD	2.5 (1.3–5.6)	3.0 (1.4–6.7)	3.6 (1.7–8.2)	< 0.01
1,2,3,4,6,7,8-HpCDD	2.1 (1.1–3.8)	3.0 (1.7–5.5)	4.5 (2.4–8.6)	< 0.01
OCDD	1.9 (1.1–3.5)	3.0 (1.7–5.5)	3.7 (2.0–6.9)	< 0.01

PCDFs				
2,3,4,7,8-PeCDF	3.8 (1.7–9.8)	4.1 (1.8–11)	5.3 (2.2–14)	< 0.01
1,2,3,6,7,8-HxCDF	1.2 (0.40–3.7)	2.9 (1.3–7.9)	2.8 (1.2–8.0)	0.06

PCBs				
PCB-126	2.5 (1.1–5.6)	4.9 (2.4–11)	9.1 (4.1–21)	< 0.01
PCB-169	1.7 (0.78–4.2)	1.7 (0.79–4.2)	1.4 (0.60–3.7)	0.73
PCB-105	1.9 (0.87–4.2)	4.6 (2.3–9.9)	7.3 (3.4–17)	< 0.01
PCB-114	3.1 (1.5–7.1)	3.6 (1.6–8.7)	6.4 (2.7–17)	< 0.01
PCB-118	2.5 (1.3–5.3)	3.8 (1.8–8.3)	6.5 (3.0–15)	< 0.01
PCB-123	1.8 (0.90–3.7)	3.4 (1.7–6.9)	5.9 (2.8–13)	< 0.01
PCB-156	1.5 (0.76–3.2)	1.7 (0.78–3.7)	2.0 (0.84–4.9)	0.23
PCB-157	1.3 (0.65–2.5)	1.1 (0.51–2.3)	1.2 (0.54–2.8)	0.81
PCB-167	2.3 (1.1–4.8)	2.7 (1.3–6.1)	4.1 (1.8–9.7)	< 0.01
PCB-189	0.69 (0.34–1.4)	1.1 (0.54–2.3)	0.98 (0.42–2.3)	0.79

Adjusted for age, sex, smoking habit, drinking habit, regional block, residential area, and survey year (model df = 19).
